# Supplementary material for: New Multitarget Rivastigmine–Indole Hybrids as Potential Drug Candidates for Alzheimer’s Disease
Source: Pharmaceutics. 2024 Feb 16;16(2):281. doi: 10.3390/pharmaceutics16020281 (PMC10892719; doi:10.3390/pharmaceutics16020281)
Supplement: Supplementary file 1 [file pharmaceutics-16-00281-s001.zip › pharmaceutics-2846507-supplementary.pdf]

Contents

-Figure S1. ^{1}HNMR spectra in MeOD-d_4 for the final compounds, rivastigmine-indole hybrids (**5a1**-**3**; **5b1**-**3**; **5c1**-**3**)

-Figure S2. ^{13}CNMR spectra in MeOD-d_4 for the final compounds, rivastigmine-indole hybrids (**5a1**-**3**; **5b1**-**3**; **5c1**-**3**)

-Figure S3. Redocking results with superimposition of the crystalized ligand a) donepezil (yellow, PDB code 4EY7 [27]) with the best docked pose within hAChE and b) *N*-((1-(2,3-dihydro-1*H*-inden-2-yl)piperidin-3-yl)methyl)-*N*-(2-methoxyethyl)-2-naphthamide (yellow, PDB code 4TPK [28]) with the best docked pose within hBChE.

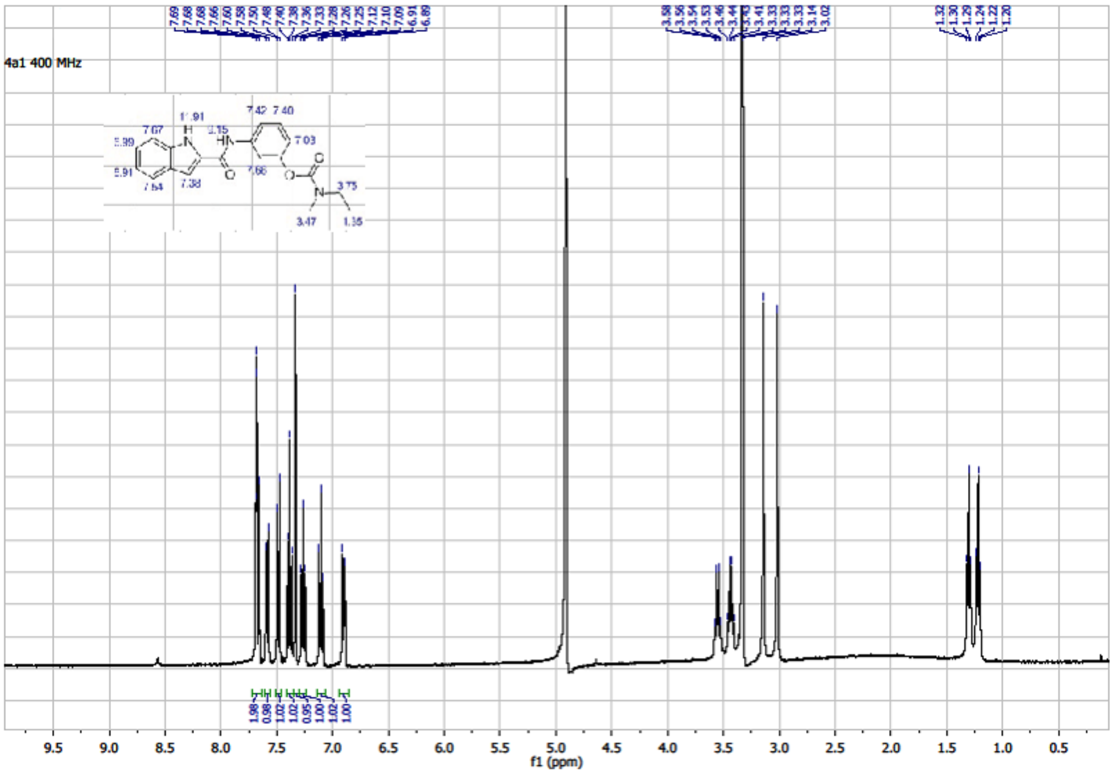

5a1

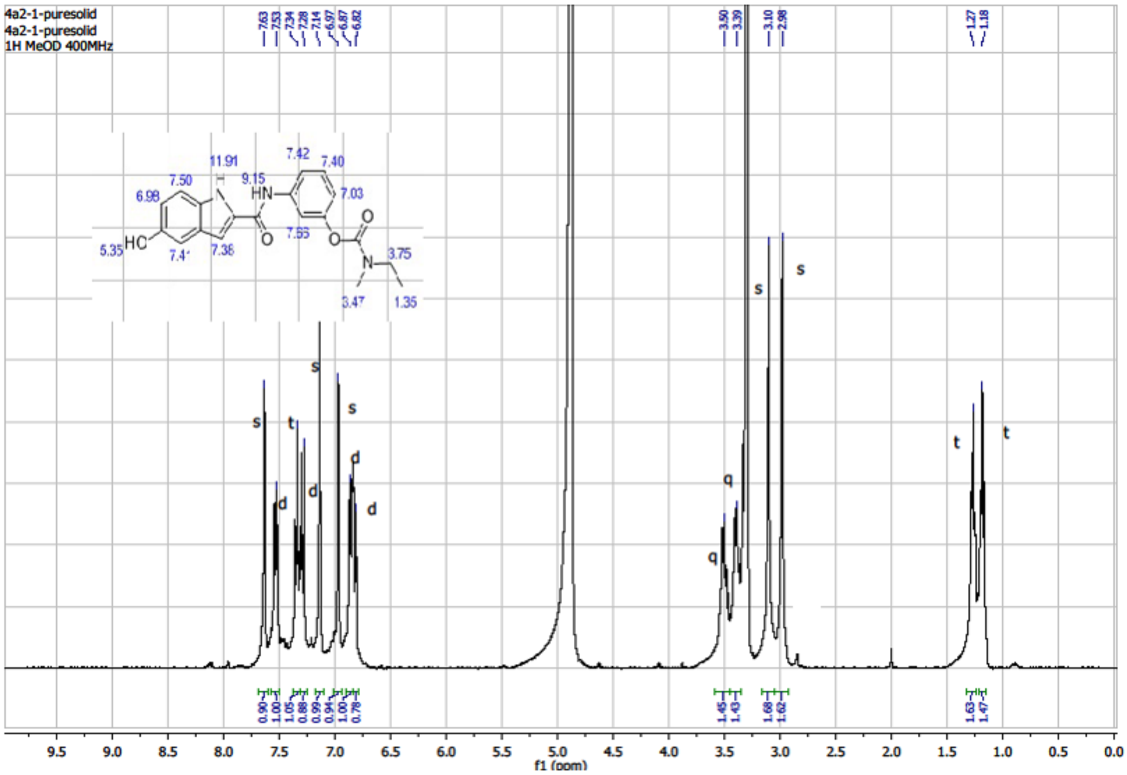

5a2

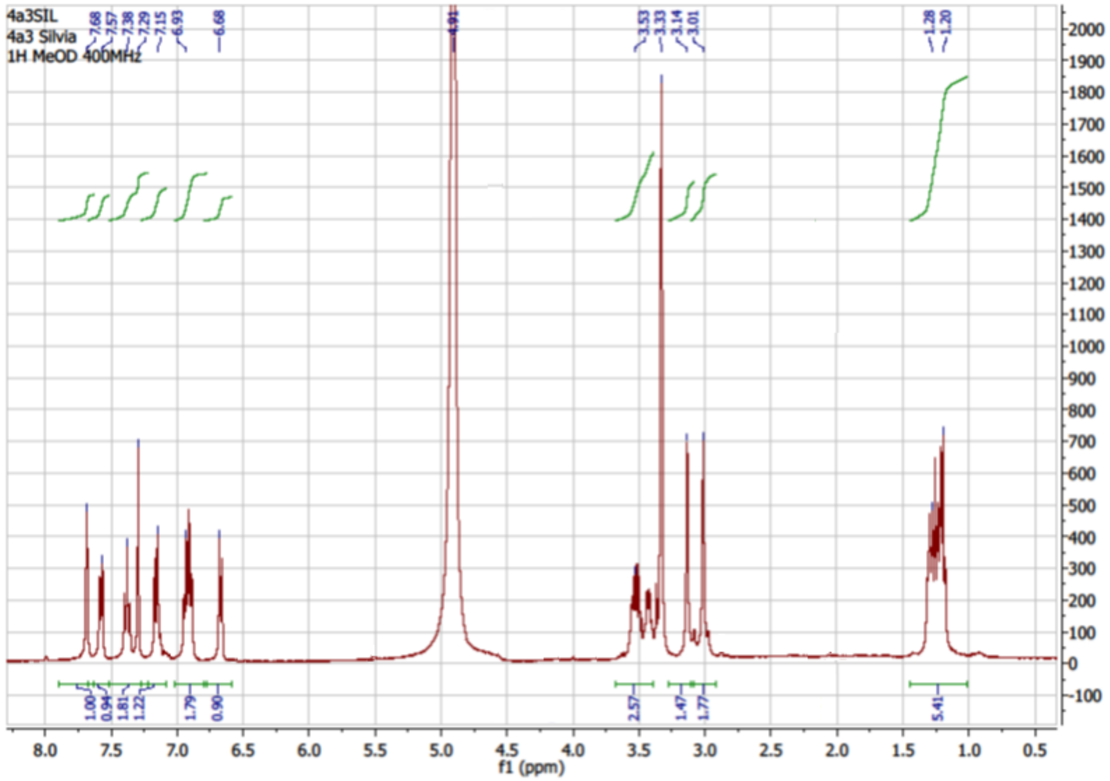
4a3SIL
4a3 Silvia
1H MeOD 400MHz
f1 (ppm)

5a3

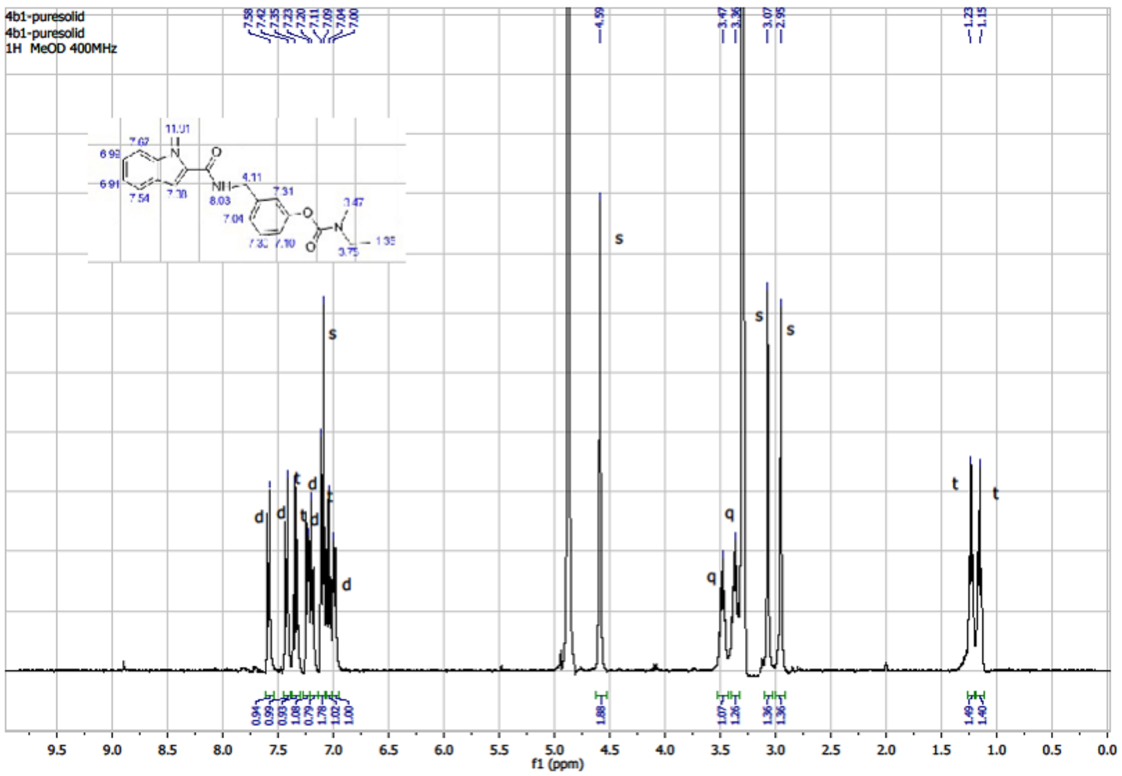
4b1-puresolid
4b1-puresolid
1H MeOD 400MHz
f1 (ppm)

5b1

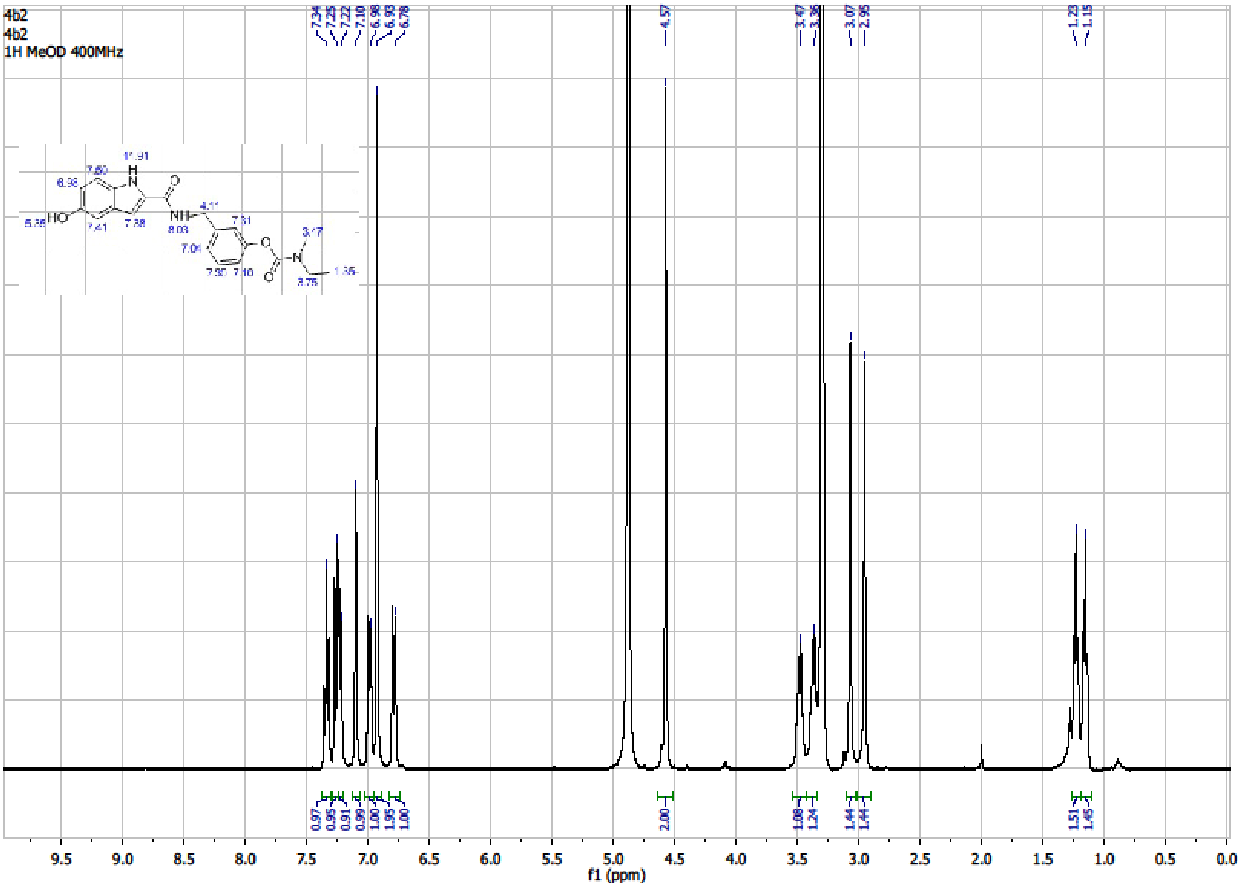
4b2
4b2
1H MeOD 400MHz
f1 (ppm)

5b2

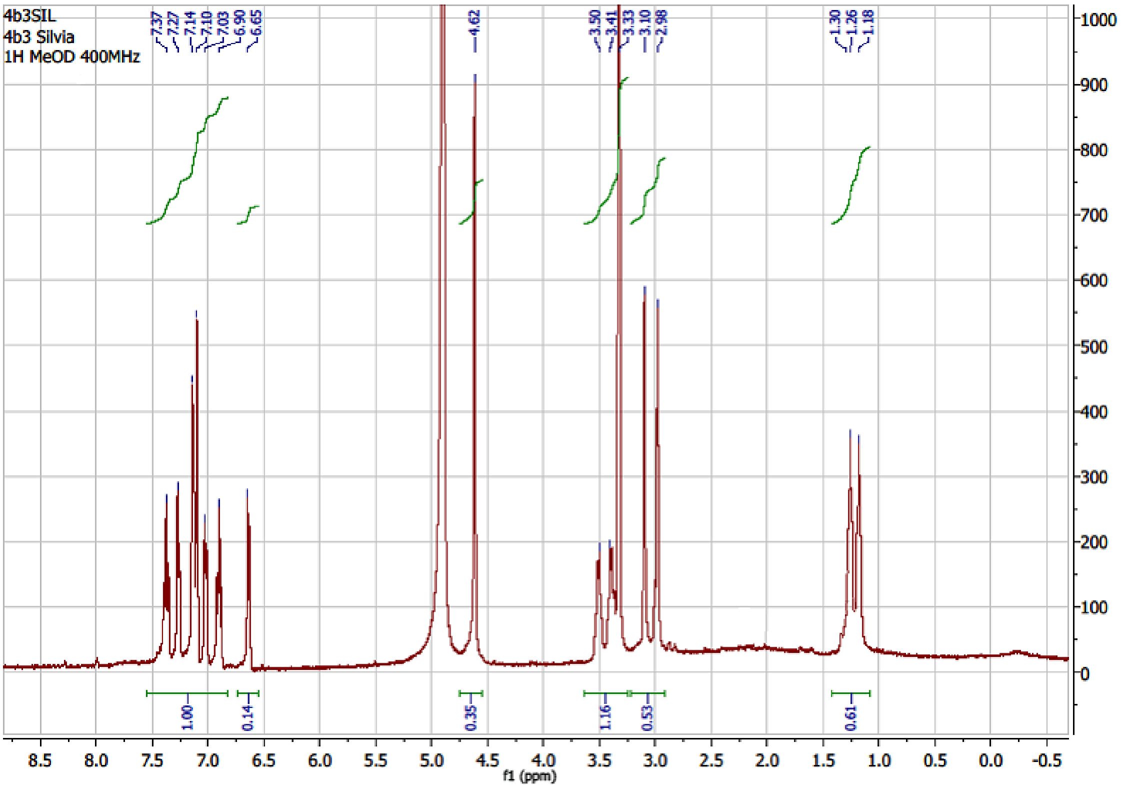
4b3SIL
4b3 Silvia
1H MeOD 400MHz
f1 (ppm)

5b3

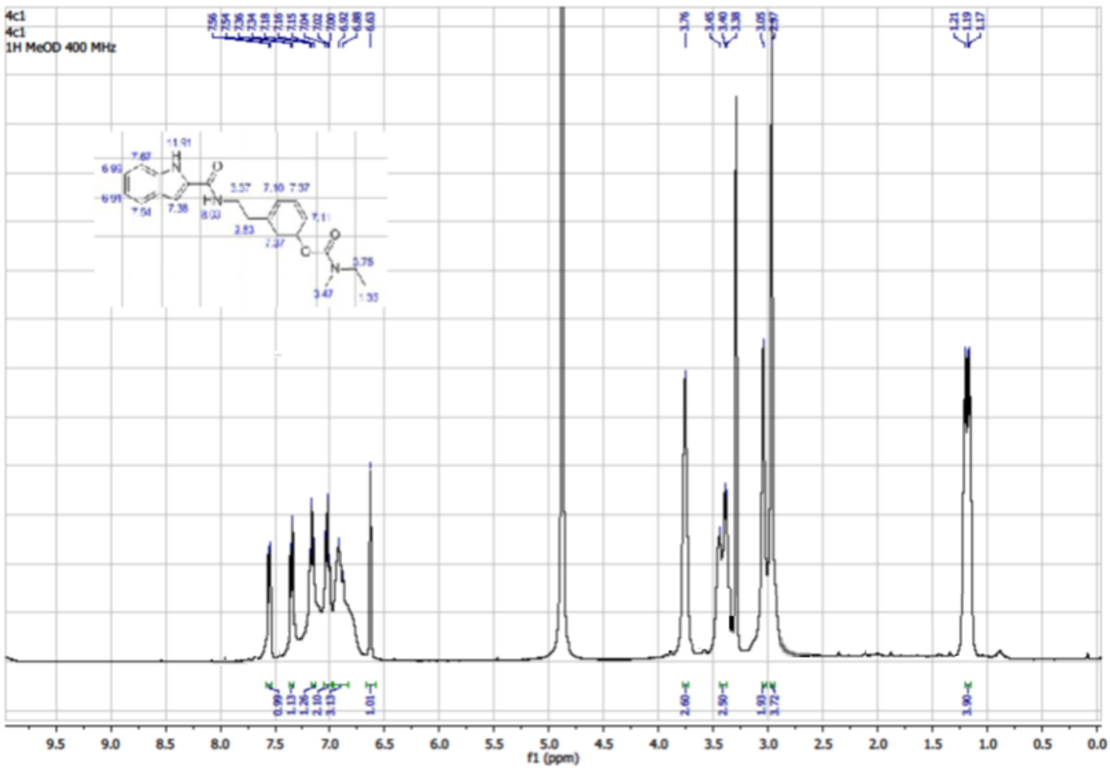

5C1

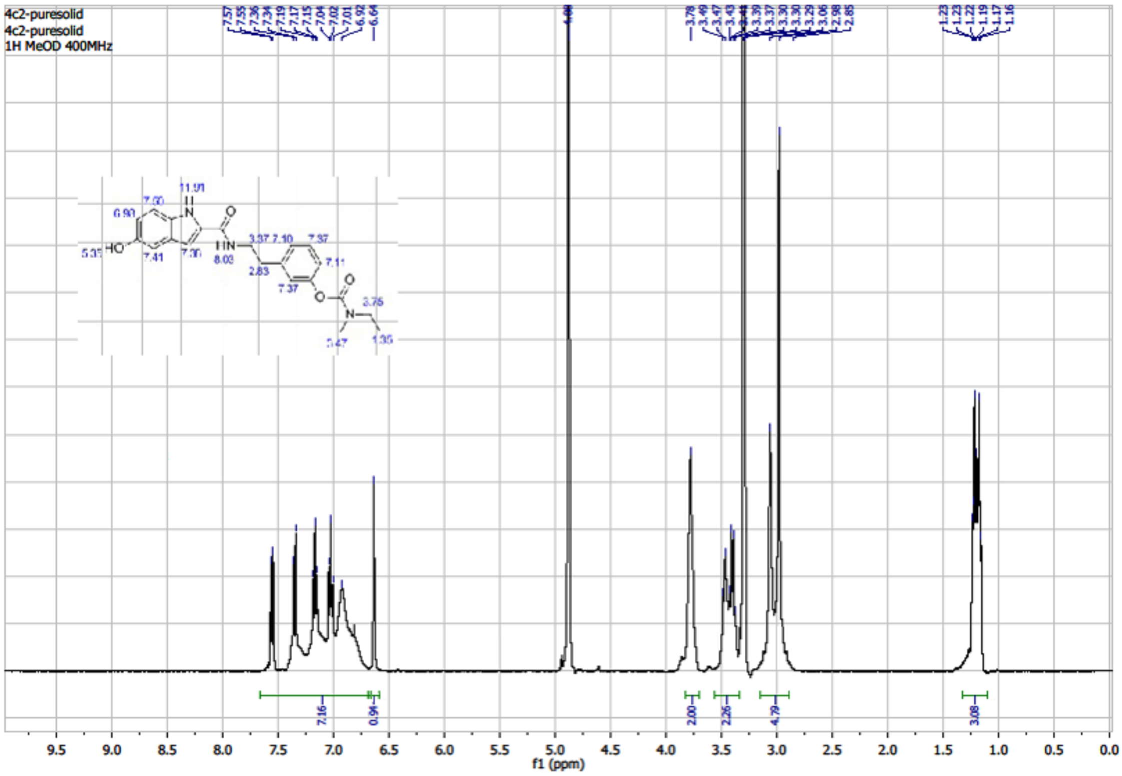

5c2

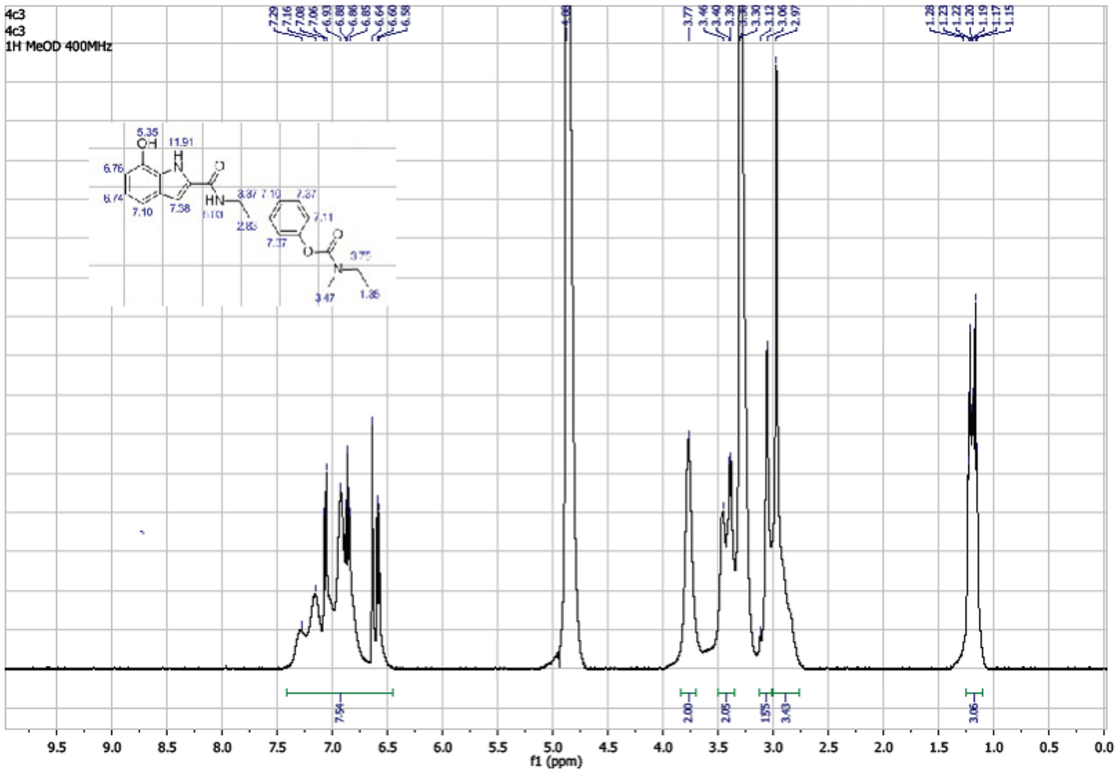

5c3

Figure S1. ^{1}HNMR spectra in MeOD-d_4 for the final products, rivastigmine-indole hybrids (**5a1-a3**; **5b1-b3**; **5c1-c3**)

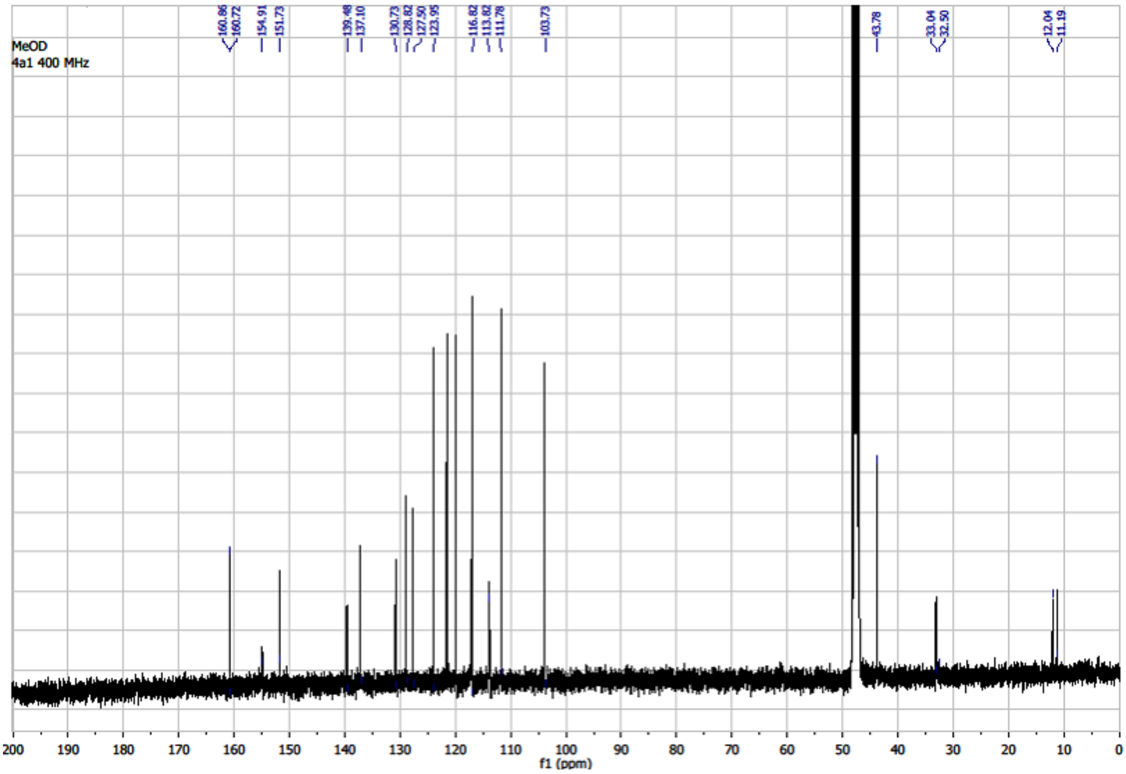
MeOD
4a1 400 MHz
160.86
160.72
154.91
151.73
139.48
137.10
130.73
128.82
127.50
123.95
116.82
113.82
111.78
103.73
43.78
33.04
32.50
12.04
11.19
f1 (ppm)

5a1

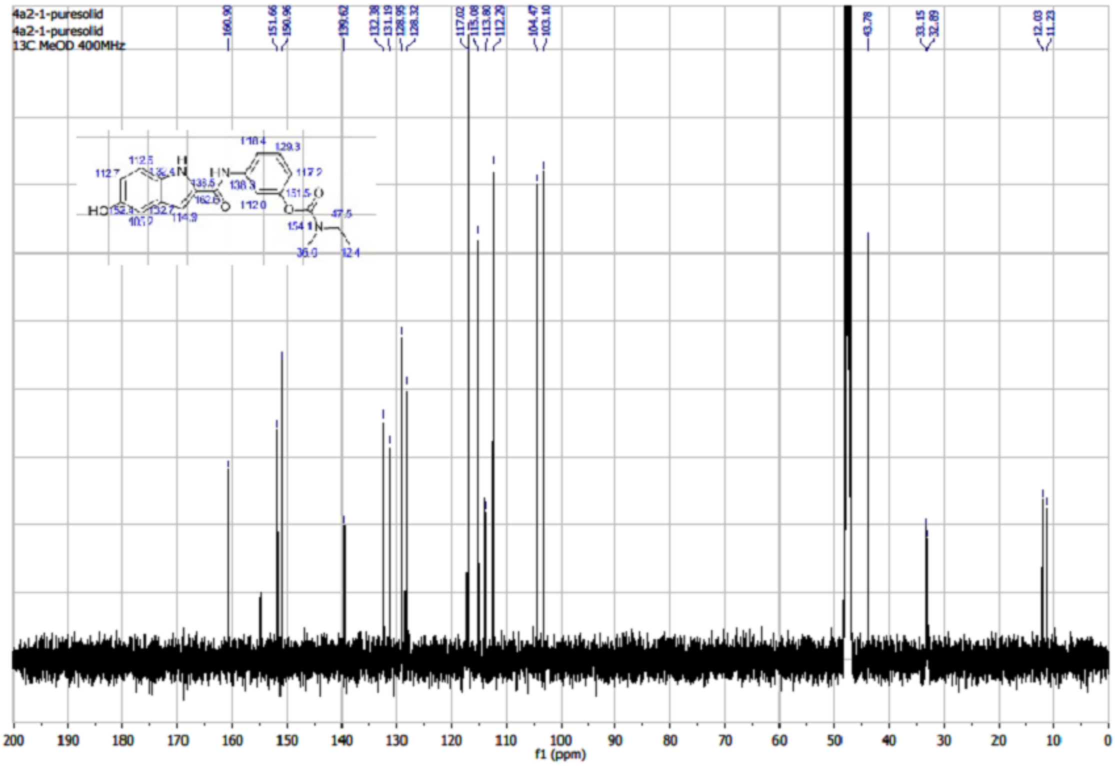
4a2-1-puresolid
4a2-1-puresolid
13C MeOD 400MHz
160.90
151.66
150.96
139.62
132.38
131.19
128.95
128.32
117.02
115.08
113.80
112.29
104.47
103.10
43.78
33.15
32.89
12.03
11.23
f1 (ppm)

5a2

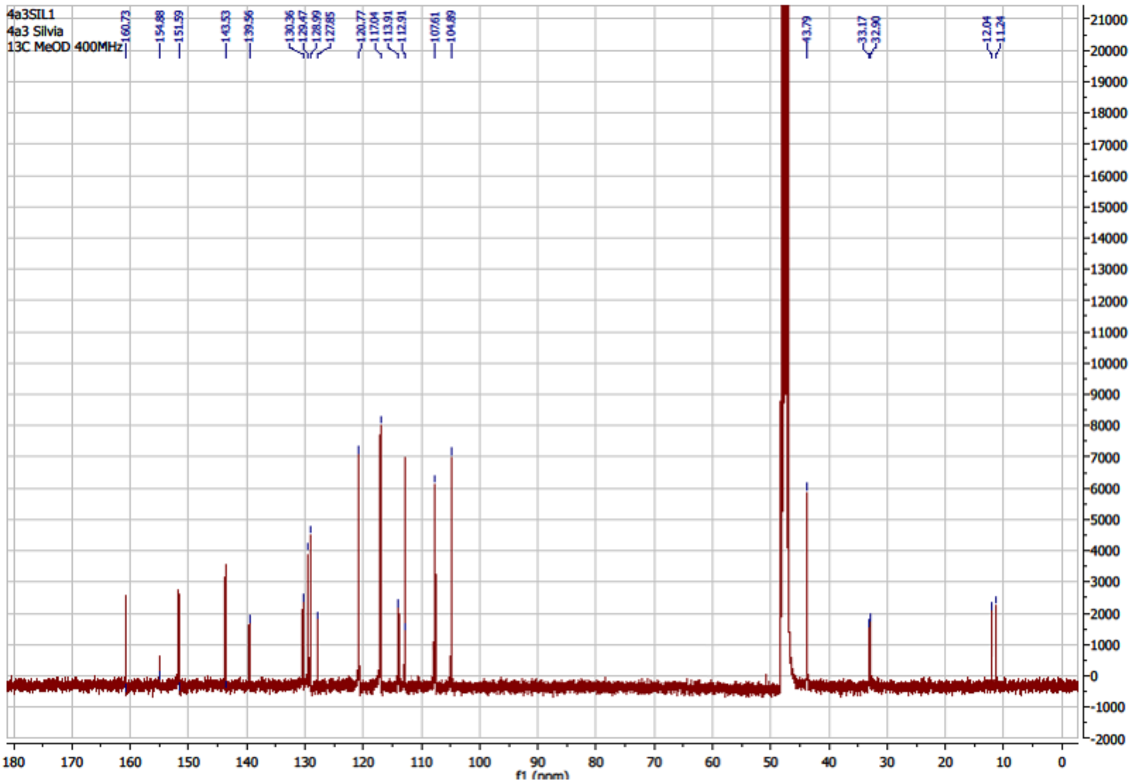
4a3SIL1
4a3 Silvia
13C MeOD 400MHz
f1 (ppm)

5a3

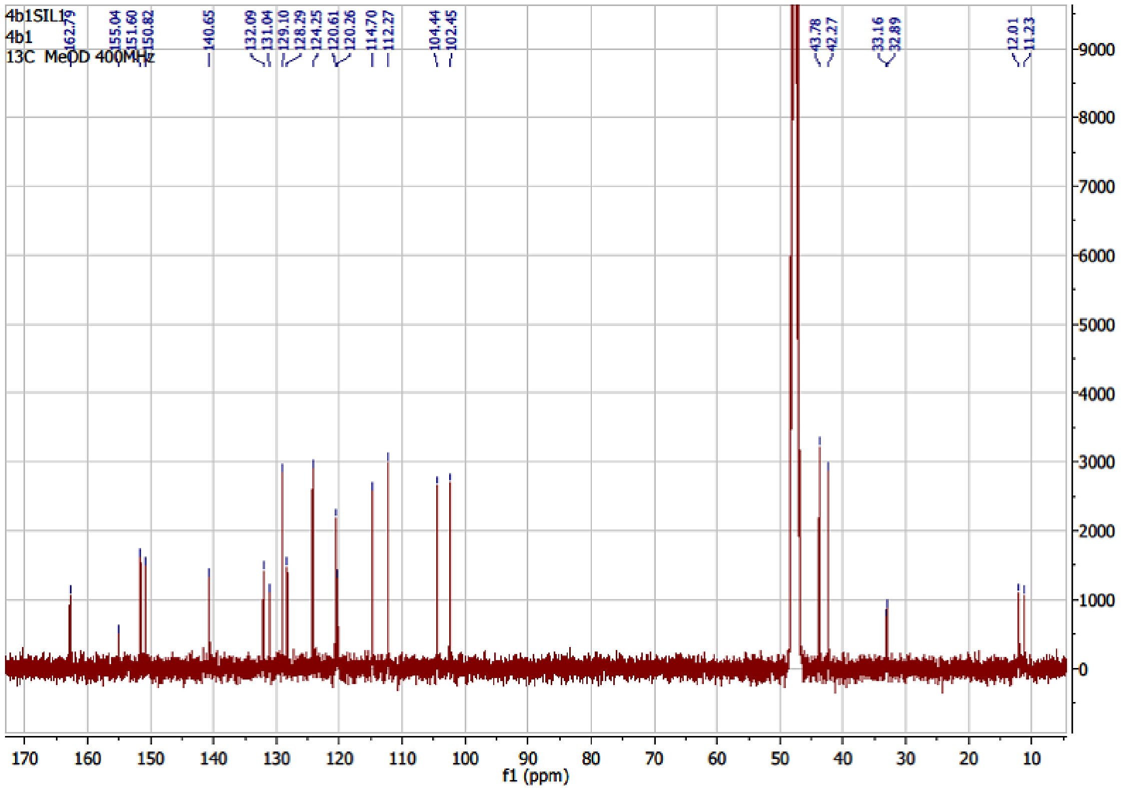
4b1SIL1
4b1
13C MeOD 400MHz
f1 (ppm)

5b1

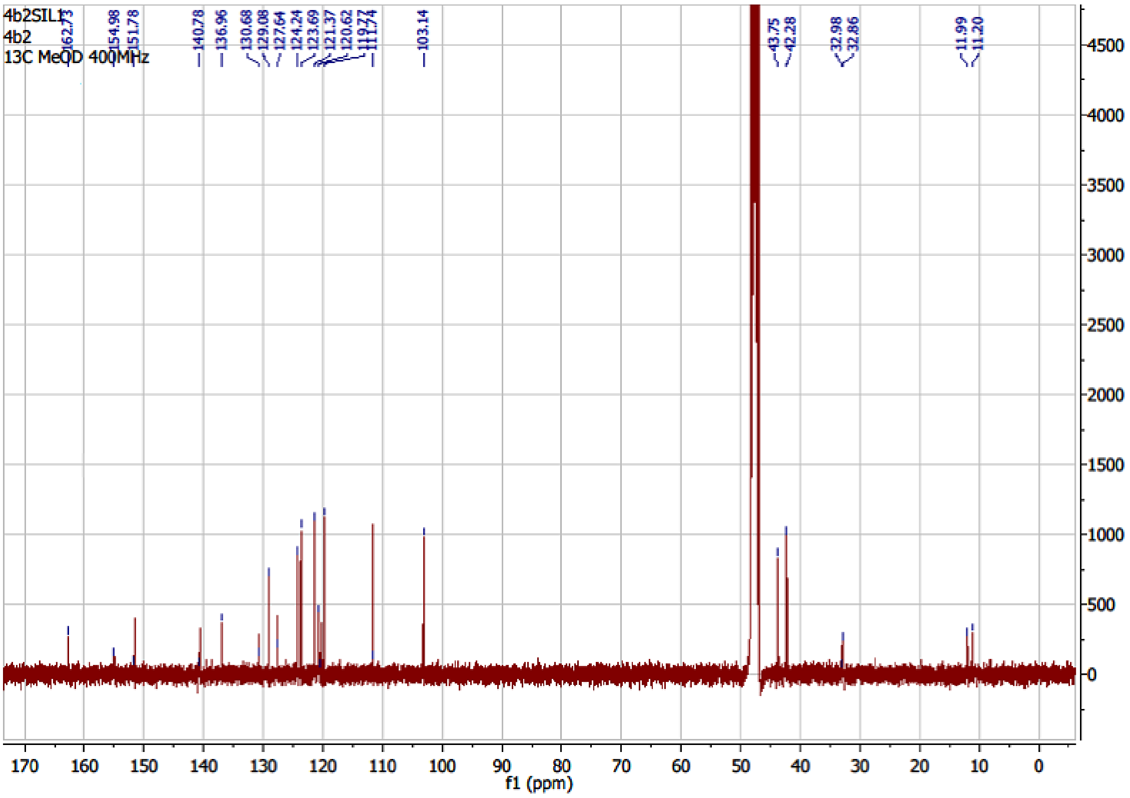

5b2

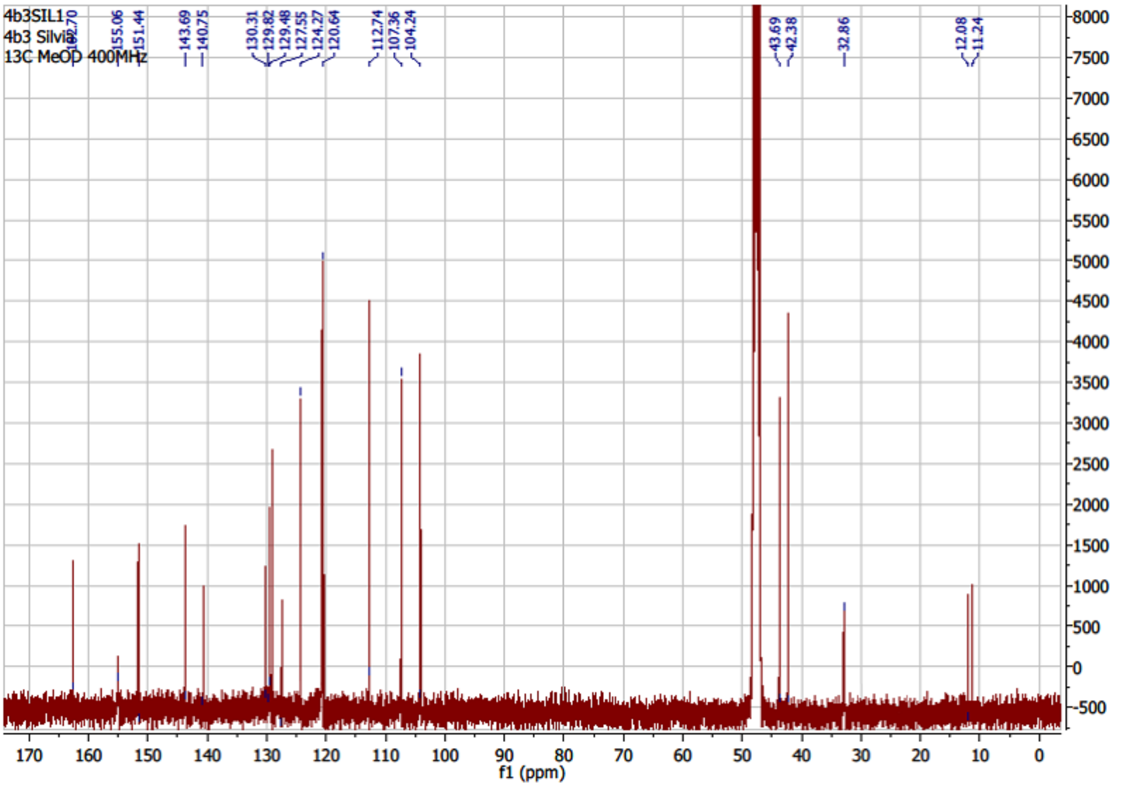

5b3

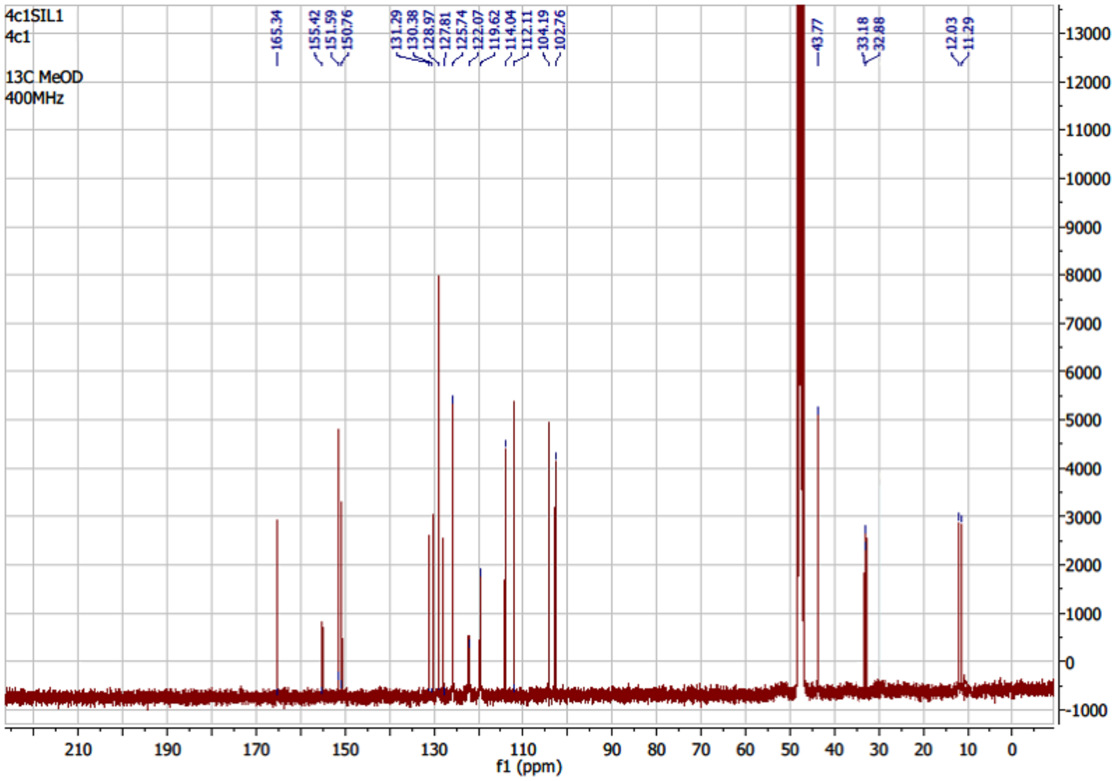
4c1SIL1
4c1
13C MeOD
400MHz
165.34
155.42
151.59
150.76
131.29
130.38
128.97
127.81
125.74
122.07
119.62
114.04
112.11
104.19
102.76
43.77
33.18
32.88
12.03
11.29
f1 (ppm)

5c1

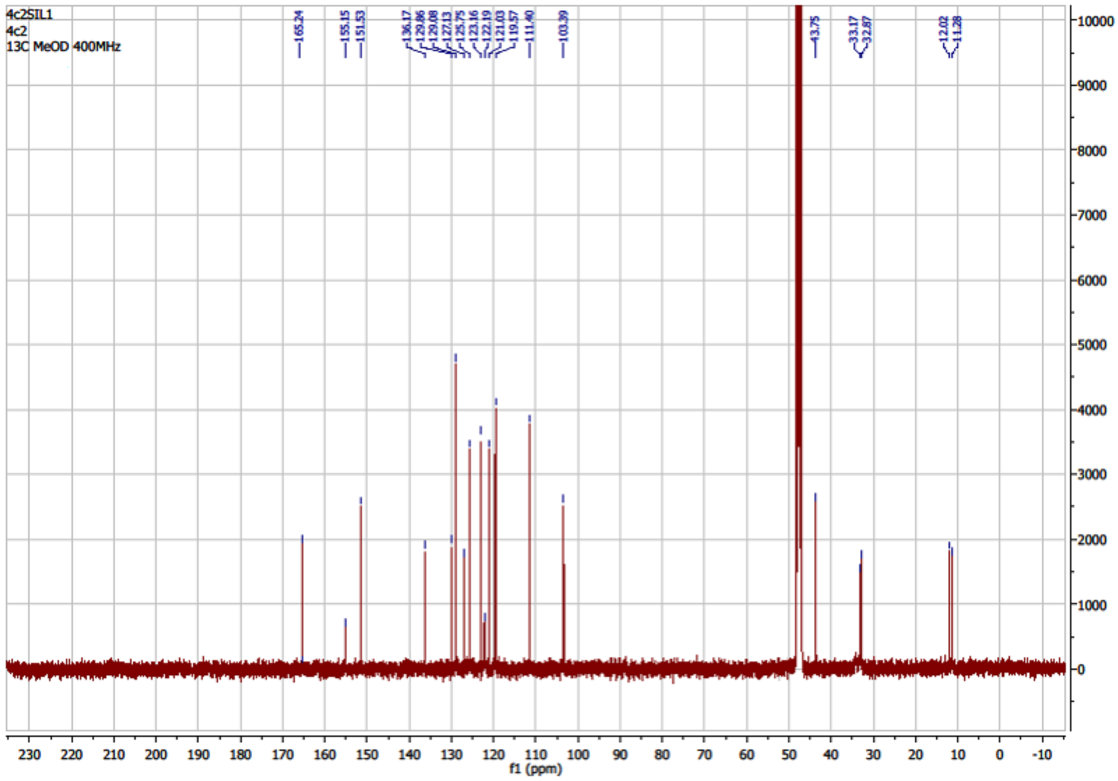
4c2SIL1
4c2
13C MeOD 400MHz
165.24
155.15
151.53
136.17
129.86
129.08
127.13
125.75
123.16
122.19
121.03
119.57
111.40
103.39
43.75
33.17
32.87
12.02
11.28
f1 (ppm)

5c2

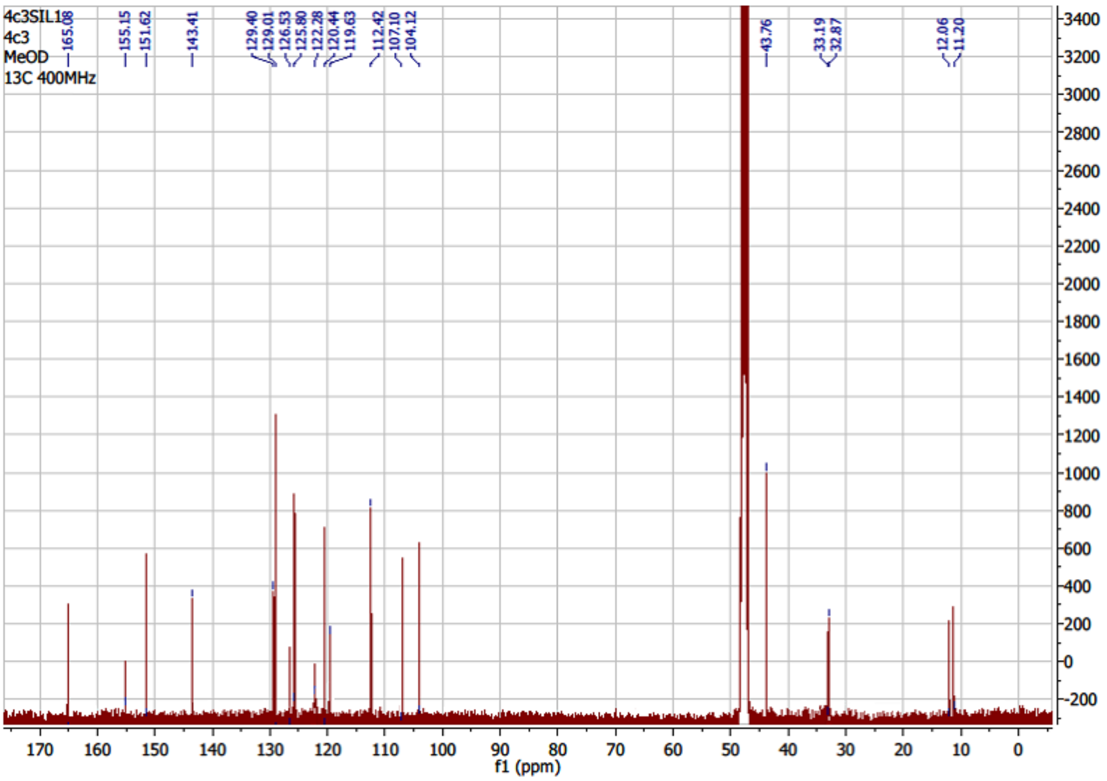

5c3

Figure S2. ^{13}CNMR spectra in MeOD-d_4 for the final products, rivastigmine-indole hybrids (**5a1-a3**; **5b1-b3**; **5c1-c3**)

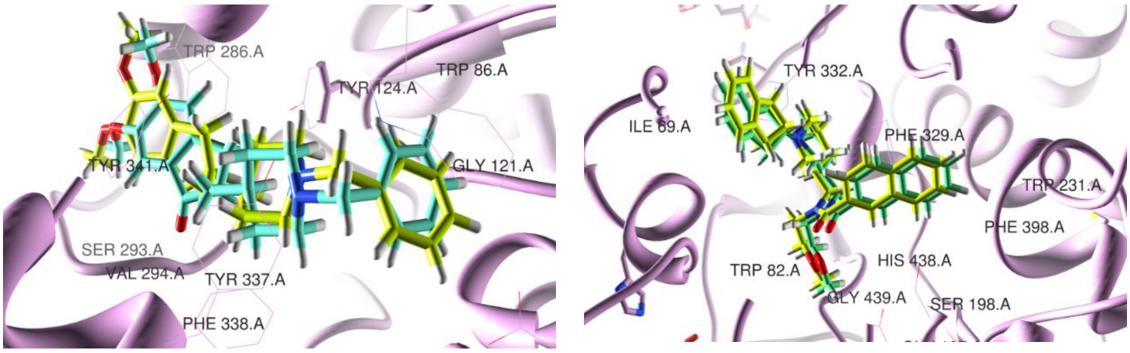

a) b)

Figure S3. Redocking results with superimposition of the crystalized ligand a) donepezil (yellow, PDB code 4EY7 [27]) with the best docked pose within hAChE and b) *N*-((1-(2,3-dihydro-1*H*-inden-2-yl)piperidin-3-yl)methyl)-*N*-(2-methoxyethyl)-2-naphthamide (yellow, PDB code 4TPK [28]) with the best docked pose within hBChE.

Table S1. Goldscore scoring function values obtained for the RIV-IND hybrids by Gold software [31].

**5a1**	63.37	57.10
**5a2**	64.46	53.65
**5a3**	63.41	56.43
**5b1**	69.36	62.81
**5b2**	68.08	60.59
**5b3**	68.63	60.53
**5c1**	71.28	64.55
**5c2**	72.82	64.82
**5c3**	69.21	65.88
Rivastigmine	47.07	44.28
